# Brodie’s Surgical Lectures

**Published:** 1845-11

**Authors:** 


### PRACTICAL MEDICINE, &c.

We would invite the careful attention of our surgical readers to the following observations from the lectures of Sir Benjamin Brodie, now in course of publication. Having space for but a small part of his observations, we are obliged to refer to the work itself for further information. In the mean time, by directing attention to the subject of Hysterical affections, simulating scrofulous diseases of the hip joint, we hope to render a service to the profession, perhaps also to some patients laboring under the disease.

The liability to hysteria is, in fact, among females, one of the severest penalties of high civilization. It is among those who enjoy what are supposed to be the advantages of affluence and an easy life that we are to look for cases of this description, not among those who, fulfilling the edict of the Deity, "eat their bread in the sweat of their face." I do not hesitate to declare, that among the higher classes of society, at least four-fifths of the female patients, who are commonly supposed to labour under diseases of the joints, labour under hysteria, and nothing else.

Frequently the symptoms are referred to the hip-joint. They then have a considerable resemblance to those of diseases in the bones or cartilages, yet a minute examination of the case will rarely leave you in doubt as to your diagnosis.

There is pain in the hip and knee, which is aggravated by pressure and the motion of the limb, and the patient often lies fixed in one position on the bed or sofa. You will say, "are not these indications of a diseased hip-joint?" But observe further. The pain is not in general fixed in any one part: it belongs to the whole limb. The patient winces, and sometimes screams, when you make pressure on the hip, but she does the same if you make pressure on the ilium, or on the side as high as the false ribs, or on the thigh, or even on the leg as low as the ankle; and everywhere the morbid sensibility is chiefly in the integuments. If you pinch the skin, lifting it at the same time off the subjacent parts, the patient complains more than when you forcibly squeeze the head of the thigh-bone into the socket of the acetabulum. As her attention is more directed to the examination, so the pain, which she suffers from it, is aggravated; and if her mind be occupied in conversation, she will scarcely complain of that, which would have occasioned torture otherwise. There is no wasting of the *glutæi* muscles, and no flattened appearance of the nates; and the aspect of the patient is different from that you would expect to find if the bones and cartilages of a joint were in a state of ulceration. Neither are there those peculiar and painful startings of the limb at night, attended often with frightful dreams which mark the existence of the last disease. The pain will sometimes prevent the patient falling asleep, but, if once asleep, she sleeps soundly for many successive hours; and this state of things may continue for weeks or months, or even for years, without leading to abscess, or any further ill consequences. There may be a suspicion of abscess, (I have known this in a great number of instances) but the suspicion is never realized. Sometimes there is a general tumefaction of the thigh and nates, the consequence either of a turgid state of the small vessels, or of an effusion into the cellular texture (I suppose of the former, as the parts do not *pit*, or remain indented after pressure); but this is entirely different from the swelling of an abscess. In a few rare instances there is a more defined and circumscribed swelling, but still it is altogether different from that of abscess. There is no perceptible fluctuation, and I can compare it to nothing better than a wheal of urticaria of unusual magnitude. A careful examination will always enable you to distinguish these swellings from abscess. For the satisfaction of others,

I have sometimes made a puncture with a grooved needle, or some other convenient instrument, the introduction of which would have detected matter, if matter had existed.

I have said that, in these cases, there is no wasting of the glutæi muscles, and no flattened appearance of the nates. It is, however, not uncommon to find much alteration in the figure of the parts, of another kind; namely, a bulging of the pelvis posteriorly, at the same time that it is elevated, on the side of the disease, so as to make an acute, instead of a right angle, with the column of the vertebræ. Of course, under these circumstances, the limb is apparently shortened, and when the patient stands erect, the heel does not come in contact with the ground. A superficial observer may be led to believe that there is an actual dislocation of the hip; and, indeed, it requires a careful examination to enable the surgeon to understand that all this strange distortion is but the result of the predominant action of certain muscles, and of a long-continued indulgence in an unnatural position.

When the symptoms are referred to the knee, they bear a near resemblance to those which have been just described. There is great tenderness of the joint; but the patient suffers more from pinching of the skin than pressure, and the morbid sensibility extends for some distance up the thigh, and down the leg, perhaps as low as the foot and ankle. She suffers less from an examination when the attention is fixed on other matters than when it is directed to the affected parts; and she does not usually complain when pressure is made on the heel, so as to press the articulating surface of the tibia against that of the femur, provided that care be taken at the same time to produce no motion of the joint. In most instances the leg is kept extended on the thigh, whereas, in cases of real disease in the knee joint, it is usually a little bent. The symptoms may continue in this case, also, without any material alteration for an indefinite time; for weeks, or months, even for years, the joint retaining its natural size and figure: but occasionally a slight degree of tumefaction is observable, especially on the anterior part, over, and on each side of, the ligament of the patella. The tumefaction is not to be confounded with a general enlargement of the joint, by which surgeons are frequently perplexed and misled, the result, not of the disease, but of the remedies employed. I refer to cases which have been misunderstood, and mismanaged by the application of blisters, issues, and a succession of various counter-irritants.

What I have now stated may be sufficient to enable you to understand the nature of the symptoms which you may expect to find where these hysterical affections occur in the other joints of the extremities. The following observations are equally applicable to all these cases, and while they are necessary to complete the history, will be found of use in enabling you to form a correct diagnosis.

The patients thus affected are, for the most part, not much above the age of puberty.

In many instances they labour under some irregularity with respect to menstruation; while in others this function is in no respect different from what it is under circumstances of perfect health.

Those who labour under habitual coldness of hands, have a weak small pulse, and afford other indications of a feeble circulation, are more liable than others to suffer in this manner; yet occasionally we find these symptoms existing in combination with a florid countenance and a sufficient developement of animal heat.

In some instances the joint to which the symptoms are referred, and even the whole limb, is affected with a remarkable alternation of heat and cold. Thus in the morning the limb may be cold, and of a pale or purple color, as if there were scarcely any circulation in it; while towards the afternoon it becomes warm, and in the evening is actually hot to the touch, with the vessels turgid and the skin shining. This state of things is often a source of serious alarm to the patient, and even to the medical attendant, but I never knew it to be followed by any ill consequences.

The majority of the patients thus affected exhibited other proofs of their liability to hysteria. Sometimes they have been subject to the usual paroxysms of hysteria, which have ceased on the local symptoms showing themselves; and a recurrence of the former has been followed by an abatement of the latter, or by complete recovery from them.

Not unfrequently the origin of these symptoms may be traced to a severe illness, which has left the patient in a state of great physical exhaustion; at other times they are as clearly to be attributed to some moral having a depressing influence on the constitution. In like manner the agency of moral causes, especially of those which compel the patient to make much physical exertion, often leads to her recovery. But we must not be led by this last-mentioned circumstance to adopt the harsh conclusion, that these symptoms exist only in those who are of a fanciful and wayward disposition. Young women of the highest moral qualities, and of the strongest understanding, are not exempt from these maladies; but it must at the same time be acknowledged that a cure is more easily attained in them than it is in others.

Although there are none of those painful and involuntary startings of the limbs which occur in combination with caries of the joints, spasmodic actions of the muscles of the limbs are not uncommon in the cases of which I am now speaking. In some instances convulsive motions of the limbs are produced, by pinching, or even lightly touching the integuments. These bear no very distant resemblance to the movements of chorea; and it is worthy of notice, that they do not occur if it can be managed, at the same time, that the attention of the patient should be otherwise directed. I have also known them to take place independently of any manifest exciting cause. In some cases which have fallen under my observation, the limb was at irregular periods violently agitated, so as almost to throw the patient off her couch.

In these cases there is always a sense of weakness in the limb, which for obvious reasons becomes aggravated in proportion as the muscles have been for a longer time in a state of inaction. While the pain and morbid sensibility of the joint are gradually subsiding, the sense of weakness increases, until at last it is the predominant symptom. Under these circumstances the patient often says, "I have no pain, but I cannot walk, because the limb is so weak." Weakness of the muscles, however, is not the only circumstance which interferes with the speedy recovery of the use of the limb in these cases. The tunics of the small blood vessels, when the limb has been long kept in the horizontal posture, seem to partake of the condition of the muscles; and when the foot is first put to the ground, the skin assumes in consequence, a red color, sometimes amounting to a purple hue, as dark as that which, when limited to a particular spot, is often the precursor of a vesication.

The symptoms which have been described for the most part come on gradually. In the majority of cases they subside gradually also; but sometimes it is otherwise, and they vanish all at once without any evident cause. For example: in the year 1834 I was consulted respecting a young lady labouring under a well-marked hysterical affection, simulating disease of the hip-joint. As she was not a resident of London I had no opportunity of watching the progress of the case, but I have lately received the following account of it from Dr. Mortimer, the surgeon at Haslar Hospital:—Her symptoms had continued nearly unaltered for nearly two years, when one night, on turning herself in bed, she said that she had a feeling as if something had given way in her hip, and from that moment she was quite well.

Another young lady was brought to London for my opinion in October, 1833. She also was supposed to labour under a disease of the hip-joint. After a careful examination of her case, I was satisfied that it was one of hysterical affection, and that there was no actual disease of the joint. I recommended her to leave her couch, to which she had been confined, and to take exercise, especially on horseback. Being a sensible and well disposed person, she followed this advice, in spite, I doubt not, of a good deal of inconvenience in the first instance. After the lapse of a year, I received from her father the following statement respecting her:—"In pursuance of your advice, she began to use the limb more freely, but with little alteration as to pain and lameness until about six weeks ago, when, by a fall of the donkey on which she was riding, she was thrown over the animal's head, standing on the foot of the lame limb, with her weight upon it. She felt immediately what she describes as a sudden snap, as if something near the joint had given way. This was attended with a violent acute pain, which, however, lasted only a short time. She was replaced on the donkey, and rode home, a distance of four miles. To her great surprise, the former habitual pain had entirely discontinued, and there has been no return of it since. She was able to walk up and down stairs without difficulty or pain, and now walks a considerable distance, using the one leg as freely and as well as the other. Her general health is improving rapidly, although she is still weak. There has been no hysterical fit since the accident; in short, the cure has been complete." However, the cure was not permanent. Three months afterwards the complaint recurred, having the same character as formerly, except that it was not now combined, as it had been in the previous attack, with other hysterical symptoms. She was at this time on the continent, and I have not heard the result of the case.

I have hitherto described these cases as if the symptoms were peculiar to the female sex; but it is not so in reality; I have known several (though by comparison certainly rare) instances of males being affected in the same manner. I employ the term hysteria because it is in common use, and because it would be inconvenient to change it for another; but the etymology of it is undoubtedly calculated to lead to a great misapprehension with respect to the pathology of that disease. It belongs, not to the uterus, but to the nervous system; and there is no one who is much engaged either in medical or in surgical practice, who will not be able to bear testimony to the accuracy of Sydenham's observation on this subject:—"*Quinimmo non pauci ex iis viris qui vitam degentes solitarum, chartis solent impallescere eodem morbo tentantur.*"

